# Interactions of Potential Anti-COVID-19 Compounds with Multispecific ABC and OATP Drug Transporters

**DOI:** 10.3390/pharmaceutics13010081

**Published:** 2021-01-09

**Authors:** Ágnes Telbisz, Csilla Ambrus, Orsolya Mózner, Edit Szabó, György Várady, Éva Bakos, Balázs Sarkadi, Csilla Özvegy-Laczka

**Affiliations:** 1 Institute of Enzymology, ELKH Research Centre for Natural Sciences, Magyar Tudósok krt. 2, 1117 Budapest, Hungary; telbisz.agnes@ttk.hu (Á.T.); mozner.orsolya@ttk.hu (O.M.); szabo.edit@ttk.hu (E.S.); varady.gyorgy@ttk.hu (G.V.); bakos.eva@ttk.hu (É.B.); 2 SOLVO Biotechnology, Irinyi József Street 4-20, 1117 Budapest, Hungary; ambrus@solvo.com; 3 Doctoral School of Molecular Medicine, Semmelweis University, Tűzoltó u. 37-47, 1094 Budapest, Hungary; 4 Department of Biophysics and Radiation Biology, Semmelweis University, Tűzoltó u. 37-47, 1094 Budapest, Hungary

**Keywords:** anti-COVID-19 agents, repurposed drugs, APP-Binding Cassette (ABC) transporters, OATP transporters, in vitro functional studies

## Abstract

During the COVID-19 pandemic, several repurposed drugs have been proposed to alleviate the major health effects of the disease. These drugs are often applied with analgesics or non-steroid anti-inflammatory compounds, and co-morbid patients may also be treated with anticancer, cholesterol-lowering, or antidiabetic agents. Since drug ADME-tox properties may be significantly affected by multispecific transporters, in this study, we examined the interactions of the repurposed drugs with the key human multidrug transporters present in the major tissue barriers and strongly affecting the pharmacokinetics. Our in vitro studies, using a variety of model systems, explored the interactions of the antimalarial agents chloroquine and hydroxychloroquine; the antihelmintic ivermectin; and the proposed antiviral compounds ritonavir, lopinavir, favipiravir, and remdesivir with the ABCB1/Pgp, ABCG2/BCRP, and ABCC1/MRP1 exporters, as well as the organic anion-transporting polypeptide (OATP)2B1 and OATP1A2 uptake transporters. The results presented here show numerous pharmacologically relevant transporter interactions and may provide a warning on the potential toxicities of these repurposed drugs, especially in drug combinations at the clinic.

## 1. Introduction

During the COVID-19 pandemic, based on in vitro experimental studies, a number of potential antiviral drugs have been proposed for the clinic. These potential treatments were rapidly brought to the attention of the medical community and the general public by the media, while in many cases, drug evaluation agencies could not properly investigate the pharmacokinetics, potential risks, and benefits. Despite this, clinicians and thousands or even millions of lay people started to compassionately use the advocated off-label compounds. The most notorious example is the wide range off-label use of the antimalarial agents **chloroquine** and **hydroxychloroquine**, in some cases together with zinc or the wide-spectrum antibacterial agent azithromycin. Chloroquine (CQ) and the less toxic analog hydroxychloroquine (HCQ) are efficient antimalarial drugs, increasing the endosomal pH in both the parasites and the host cells. HCQ is also clinically used in autoimmune diseases [1]. However, these compounds were previously found to have major toxicities, especially by prolonging the cardiac QT interval or causing hypoglycemia. CQ and HCQ were both reported to be moderate inhibitors of CYP2D6 and the ABCB1/Pgp transporter. Interestingly, azithromycin has a similar toxicity to CQ and HCQ to prolong the QT interval [2].

The potential use of CQ and HCQ in COVID-19 was initiated by in vitro studies, in which both compounds inhibited the fusion of SARS-CoV-2 with the cell membranes, and reduced ACE2 receptor glycosylation (the binding site of SARS-CoV-2) and the transfer of SARS-CoV-2 from early endosomes to lysosomes [3,4,5]. However, whilst CQ and HCQ—also in combinations with azithromycin—have been examined in several clinical trials, their use has not been approved by the EMA or FDA for the treatment of COVID-19. A recent statement of the NIH COVID-19 Treatment Guidelines Panel (NIH-CTGP) strongly advises against the use of CQ or HCQ because of inefficiency and the cardiac complications [6,7].

Another proposed anti-COVID-19 agent—**ivermectin**—is a broad-spectrum anti-parasitic agent and widely used to treat neglected tropical diseases. The target of the antiparasitic action of ivermectin is a glutamate-gated chloride channel and a GABA receptor, specific for some invertebrates. However, in mammals, ivermectin may also inhibit GABAergic neurotransmission by promoting the release of GABA and acting as a GABA receptor agonist. This potentially neurotoxic drug is only absorbed in humans in a very small fraction, and this low-level ivermectin absorption is mainly caused by active extrusion in the intestine by the ABCB1/Pgp transporter. ABCB1, and probably other ABC transporters (especially ABCG2) in the blood–brain barrier (BBB), also have a significant role in protecting the mammalian central nervous system (CNS) against toxic ivermectin penetration. In mouse Pgp-knock-out models, in natural Pgp-knock-out Collie dogs, and also in some humans with low-level ABCB1/Pgp expression, ivermectin exerts major neurotoxicity [8,9]. In addition, both ABCB1 and several other multispecific transporters have been shown to be inhibited by micromolar concentrations of ivermectin [10,11,12], so ivermectin may influence the pharmacokinetics of several drugs or toxic compounds. Ivermectin was suggested to have anti-CoV-2 effects based on in vitro studies, indicating that this compound inhibits the importin alpha/beta-1 nuclear transporter, and thus in cell cultures, reduces the replication of various viruses [13,14,15], including SARS-CoV-2 [16,17]. However, the ivermectin plasma concentrations required to reach an in vitro antiviral efficacy (about 2–10 μM) are highly toxic, and very high oral doses would be needed for antiviral use [18]. Therefore, in spite of some anecdotal clinical results, ivermectin has not been approved for the treatment of any viral diseases, including SARS-CoV-2 (see the NIH-CTGP). Moreover, in April, 2020, FDA issued a warning not to use ivermectin to treat COVID-19 in humans.

Several approved and clinically effective HIV protease inhibitors, including **ritonavir** and **lopinavir**, based on their potential for inhibiting other viral proteases, also entered clinical trials as anti-COVID-19 agents. SARS-CoV-2 virus replication requires the cleavage of viral polyproteins, and the proteases 3CLpro and PLpro are responsible for this cleavage. In vitro, lopinavir and ritonavir were found to inhibit 3CLpro, and thus reduce SARS-CoV-2 replication [19]. However, these drugs are poorly selective and high concentrations are required to achieve in vivo inhibition [20]. Therefore, the current NIH-CTGP recommends against the use of these compounds for COVID-19 treatment, except in a clinical trial. The NIH-CTGP also warns that the lopinavir/ritonavir combination is a strong inhibitor of cytochrome CYP3A, so co-treatment with numerous drugs metabolized by CYP3A may result in increased toxicities. Ritonavir induces CYP1A2 and inhibits CYP3A4 and CYP2D6. Therefore, it may cause serious drug–drug interactions. Ritonavir and lopinavir are inhibitors of ABC multidrug transporters [21,22], both in vitro and in vivo [23], and according to studies with labeled ritonavir and lopinavir, these agents are transported substrates of ABCB1/Pgp [24,25], but not of ABCG2/BCRP [26,27].

Recently developed antiviral agents, despite having been found to be minimally effective in earlier clinical studies and not approved for general use, have also been introduced inCOVID-19 clinical trials. **Favipiravir** (Avigan) is a pyrazinecarboxamide derivative antiviral agent, administered as a prodrug in both oral and intravenous formulations. The active form is an inhibitor of the viral RNA-dependent RNA polymerase and thus reduces the viral load. Favipiravir was approved in Japan against special cases of influenza, although it was found to be ineffective in human airway cells [28]. Favipiravir degradation mostly occurs by aldehyde oxidase (AO) and xanthine oxidase. Since favipiravir was found to inhibit CYP2C8, co-administration with CYP2C8 substrates may increase some drug effects. Since favipiravir is often used together with acetaminophen, it is important to note that favipiravir inhibits acetaminophen sulfate formation. In addition, favipiravir has been shown to have a teratogenic effect in animals [29]. Currently, the use of favipiravir in treating COVID-19, despite only having limited clinical data for its efficiency in this disease, has been approved in China, India, and for emergency use in Japan, while this agent remains unapproved in Europe and the USA.

**Remdesivir** (Veklury) is a broad-spectrum antiviral medication employed for intravenous injection provided in a sulfobutylether-β-cyclodextrin (SBECD) complex. Remdesivir (RDV) itself is a prodrug, and its active triphosphate metabolite is a ribonucleotide analogue inhibitor of viral RNA polymerase. Remdesivir was developed for the treatment of hepatitis C and Ebola virus diseases, while based on initial clinical trials, it has been authorized for emergency use in COVID-19 in numerous countries, especially for patients with severe symptoms. In October 2020, FDA authorized the clinical use of remdesivir against COVID-19, while the WHO still does not support its use in this disease. Preclinical studies indicated that remdesivir is not a substrate of CYP2C8, CYP2D6, or CYP3A4, and is not “significantly” transported by ABCB1/Pgp or the organic anion-transporting polypeptide (OATP)-type drug transporters [30], while detailed studies on remdesivir–drug interactions are not yet available. Remdesivir in in vitro studies was metabolized by CYP2C8, 2D6, and 3A4, while in vivo studies indicate its metabolism by hydrolases. Remdesivir at the clinic is applied in the form of a sulfobutyl ether beta-cyclodextrin (SBECD) complex, thus, in addition to remdesivir, we have also used this cyclodextrin complex in our in vitro studies.

In order to explore the multispecific drug transporter interactions of the above repurposed drugs (see Table 1), in this investigation, we performed a detailed in vitro study on their potential interaction with several key multidrug transporters. We focused on the potential role of the transporter–drug interactions in the important tissue barriers, especially the intestinal epithelium and the blood–brain barrier (BBB) endothelial cells (see http://www.fda.gov/Drugs/DevelopmentApprovalProcess/DevelopmentResources/DrugInteractionsLabeling/ucm093664.htm#major (2017)).

The ABCB1/Pgp and ABCG2/BCRP efflux transporters are key proteins for xenobiotic extrusion in the intestine and BBB, while they are also involved in toxin and drug metabolism in the liver and kidney [31,32,33]. In addition to several endogenous substrates, including conjugated metabolites, steroids, and uric acid, there is a wide range of environmental and food-related toxic molecules actively transported by these proteins. Therefore, they are major players in drug–drug interactions. While, in most model animals, Pgp is the major drug transporter in the BBB, in primates and humans, ABCG2 seems to be the key protective transporter in this tissue [34,35,36]. In addition, ABCC1 has a role in the blood–cerebrospinal barrier to avoid CNS toxicity [37].

Organic anion-transporting polypeptides (OATPs) belong to the Solute Carrier (SLC) family and some of them (e.g., OATP1A2, 1B1, 1B3, and 2B1) are well-documented multispecific plasma membrane xenobiotic and drug transporters involved in the cellular uptake of various organic molecules (“uptake transporters”) [38]. OATP1B1 and OATP1B3 are important in liver drug metabolism, while OATP1A2 and OATP2B1 are expressed in a variety of human tissues [39]. These latter transporters are highly expressed in the intestine, while OATP1A2 is the key drug uptake transporter expressed in the BBB endothelial cells, promoting drug uptake from the blood plasma into the CNS [40,41,42]. Therefore, OATPs 1A2 and 2B1 are important players in the pharmacokinetics of numerous drugs, either being substrates or inhibitors of these transporters. Based on both in vitro and clinical data, OATP1A2 and OATP2B1 are key players in the intestinal uptake of a large variety of clinically important drugs, including statins, fexofenadine, sulfasalazine, steroids, and telmisartan, while they may be significantly inhibited by chemotherapeutics or antiviral compounds [42]. In addition, they contribute to the tissue penetration of numerous endogenous substrates, including steroid and thyroid hormones, prostaglandins, bile acids, and bilirubin [43]. Based on their important role in tissue barriers and drug pharmacokinetics, we studied the potential inhibition of the function of ABCB1, ABCG2, ABCC1, OATP2B1, and OATP1A2 by the clinically applied anti-COVID-19 agents. In these experiments, we used an array of in vitro functional transporter assays which, together, may provide important new information regarding the potential ADME-tox properties of these agents.

## 2. Materials and Methods

### 2.1. Materials

All basic laboratory reagents were obtained from Sigma Aldrich. The 5D3 antibody was a kind gift from B. Sorrentino, St. Jude Children Hospital, Memphis, USA, and remdesivir and remdesivir-SBECD were kind gifts from Lajos Szente, Cyclolab Ltd., Budapest, Hungary. Vesicular assay membranes and components were obtained from SOLVO Biotechnology, Budapest, Hungary (https://www.solvobiotech.com/services/categories/). 

### 2.2. ABC Transporter Assays

For the ABC transporter assays, we used both cell-based and membrane-based functional assays. For assaying the function of the ABC transporters in cell-based assays, we applied stable cell lines expressing the transporters ABCG2 PLB-985, ABCB1 PLB-985, and ABCC1 HL-60, and their parental lines, which lack significant ABC transporter expression [44]. The respective transport activities were assayed by measuring the cellular fluorescence of the respective transported substrates Phengreen (ABCG2) and Calcein (ABCB1 and ABCC1) [44]. For the ABCG2 transport assay, we also used HeLa cells stably expressing the wild-type or Q141K polymorphic variant of the ABCG2 protein (see below).

For studying ABCB1 and ABCC1 transport, 5 × 10^4^ parental control or transporter-containing cells were incubated with 0.25 µM Calcein-AM (C3100MP ThermoFisher Scientific, Walthman, MA, USA) in phosphate buffered saline (PBS) containing 1 g/L D-glucose (DPBS) for 40 min at 37 °C. The test compounds were applied in 0.2–50 µM, and as specific transporter inhibitors, 0.25 µM tariquidar (TQ, which was a kind gift from Dr. S. Bates (NCI, NIH)) for ABCB1 and 10 µM indomethacin (IM, I0200000, Sigma-Aldrich-Merck, St. Louis, MO, USA) were used. The cells were kept on ice until the flow cytometry measurements. All experiments were performed in triplicate and in at least three biological parallels.

For examining the function of ABCG2, 5 × 10^4^ parental control or ABCG2 transporter-expressing cells were incubated with 0.25 µM PhenGreen-SK diacetate (P14313, ThermoFisher Scientific, Walthman, MA, USA) in DPBS containing 1 µM EDTA, for 40 min at 37 °C. The test compounds were applied in 0.2–50 µM, and as a specific transporter inhibitor, 2.5 µM Ko143 (3241, Tocris Bioscience, Bristol, UK) was applied. The cells were kept on ice until the flow cytometry measurements. All experiments were performed in triplicate and in at least three biological parallels. Cellular fluorescence reflecting the transport activity was measured by an Attune Nxt cytometer (Thermo Fischer Scientific, Waltham, MA, USA) equipped with a blue (488 nm) laser. The PhenGreen (PG) or Calcein signal was detected in the BL1 channel (emission filter: 530/30 nm). Analysis of the data was carried out by the Attune Nxt Cytometer Software v3.1.2 (Thermo Fischer Scientific, Waltham, MA, USA). The inhibition of transporter activity was calculated by comparing the fluorescence in the presence of the test compound to that in the presence of a specific inhibitor providing maximum inhibition. Higher concentrations of some of the investigated compounds also altered the fluorescence of Calcein in the parental cells in an ABC transporter-independent way (parental cells had no significant ABC transporter expression, as tested by specific inhibitors), and these data were used for correction of the data obtained in ABC transporter-expressing cells. Half-maximum inhibition (IC_50_) values were calculated by using the Origin2019 software (9.6.5.169, OriginLab Corporation, Northampton, MA, USA). In addition to studies on the wild-type ABCG2 transporter, we also examined the effects of drugs on the function of a relatively frequent (present in up to 20–35% of the population) polymorphic variant—the Q141K-ABCG2 (SNP rs2231142) transporter. In these experiments, we measured Hoechst dye extrusion in HeLa cells stably expressing eGFP and the ABCG2 variants driven by the same promoter—the transgenic cells generated by the Sleeping Beauty transposon system. These cells were sorted based on similar levels of eGFP, and ABCG2 expression was examined by a monoclonal (5D3) antibody-based flow cytometry assay (Q141K-ABCG2 cell surface expression was approximately 80% of the wild-type ABCG2 expression. For details, see Zámbó et al. [45]). For the ABCG2 transporter inhibition assay, trypsinized HeLa cells expressing the ABCG2 wild-type (WT) or Q141K variant were incubated in a shaker for 20 min at 37 °C in HPMI buffer (20 mM HEPES, 132 mM NaCl, 3.5 mM KCl, 0.5 mM MgCl_2_, 5 mM glucose, 1 mM CaCl_2_ [pH 7.4]) with 0.2 µM of Hoechst 33342 (H1399, Thermo Fisher Scientific, Waltham, MA, USA) dye, in the presence of the tested drugs or 1 µM of the specific ABCG2 inhibitor Ko143 (3241, Tocris Bioscience, Bristol, UK). Following incubation, the cells were put on ice and Hoechst dye fluorescence was measured using the violet laser (405 nm) and VL1 detector on the Attune Nxt Cytometer. Cells showing similar eGFP fluorescence were gated in the case of the WT and Q141K variants. The maximum inhibition was defined for each variant as the fluorescence difference of the samples incubated with and without Ko143, respectively. All tested drugs (ivermectin, remdesivir, ritonavir, lopinavir, and Ko143) were dissolved in DMSO, and the same DMSO concentration (below 0.5%) was applied for all drug concentrations examined. Data analysis was performed using the Attune Nxt Cytometer Software v3.1.2. Two samples per condition and three biological replicates were examined in each experiment.

For studying the vesicular ATP-dependent transporter activity of the ABC transporters, we used ABCB1/Pgp or ABCG2/BCRP containing HEK-293 cell membrane vesicles, as well as ABCC1/MRP1 containing Sf9 membrane vesicles, prepared by Solvo Biotechnology. Membrane vesicles (12.5 μg protein/sample) were incubated with transporter-specific substrates: For ABCG2, 5 μM lucifer yellow (LY) as a fluorescent substrate and the radiolabeled substrate probes; for ABCB1, 1 μM N-methyl quinidine (NMQ); and for ABCC1, 0.2 µM estradiol-glucuronide (ETGB). These were employed at 37 °C for 10 min (ABCG2), 1 min (ABCB1), or 3 min (ABCC1), with or without 4 mM Mg-ATP, in a 50 μL final volume. The known reference inhibitors of the transporters (1 µM Ko143 for ABCG2, 1 µM Valspodar for ABCB1, and 200 µM Benzbromarone for ABCC1) served as controls. Each test compound was dissolved in DMSO and a 1 µL volume was added to the samples. After incubation, the samples were filtered and washed, and the substrates in the vesicles were dissolved in 10% SDS and transferred onto another plate. After the addition of a stabilizer, the fluorescence was measured in plate readers (Victor X3 and Enspire Perkin-Elmer, Waltham, MA, USA), while the activity of the radiolabeled substrate was measured by liquid scintillation counting (Perkin Elmer MicroBeta2 liquid scintillation counter, Perkin Elmer, Waltham, MA, USA). ABC transporter-related vesicular transport was calculated by subtracting uptake measured in the presence of Mg-AMP from the values measured in the presence of Mg-ATP. No significant quenching by the test compounds was observed.

ABC transporter ATPase activity was measured in Sf9 membrane vesicles containing the respective human ABC transporters [46,47,48,49,50]. For ABCG2/BCRP, the cholesterol level in the vesicles was increased to the level of mammalian cell membranes for full activity [49]. For ABCB1/Pgp 20 μg/150 μL and for ABCG2/BCRP 10 μg/150 μL, vesicles were incubated with 3 mM Mg-ATP for 25 min at 37 °C. The test drugs were dissolved in DMSO and 1 µL was added to the samples. The ABC transporter-related ATPase activity was determined and reference activators (50 µM verapamil for ABCB1 and 5 µM quercetin for ABCG2) served as controls.

### 2.3. OATP Transporter Assays

For studying the function of the multispecific uptake transporters OATP1A2 and OATP2B1, we used A431 human tumor cells overexpressing these human proteins and mock transfected A431 cells as controls, generated as previously described [47,51]. The interaction between OATPs and the potential anti-COVID-19 agents was studied by employing fluorescent dye substrates: Pyranine for OATP2B1 and sulforhodamine 101 for OATP1A2 [51,52].

Briefly, A431 cells overexpressing OATP1A2 or OATP2B1 [47] were seeded on 96-well plates, washed three times with 200 μL phosphate buffered saline (PBS, pH 7.4), and pre-incubated with 50 μL uptake buffer (125 mM NaCl, 4.8 mM KCl, 1.2 mM CaCl_2_, 1.2 mM KH_2_PO_4_, 12 mM MgSO_4_, 25 mM MES (2-(Nmorpholino) ethanesulfonic acid, and 5.6 mM glucose, pH 5.5 for OATP2B1 and pH 7.0 for OATP1A2) at 37 °C, with or without increasing concentrations of the tested compounds. The test compounds were dissolved in DMSO (maximum 0.5%), and the reaction was started by the addition of a final concentration of 20 μM pyranine (OATP2B1) or 0.5 μM sulforhodamine 101 (OATP1A2). After incubation at 37 °C for 15 min (OATP2B1) or 10 min (OATP1A2), the reactions were stopped and the cells were washed three times with ice-cold PBS. Fluorescence was measured in an Enspire plate reader (Perkin Elmer, Waltham, MA, USA) (ex/em: 403/5 and 517/5 nm (pyranine) or 586/5 and 605/5 nm (sulforhodamine 101)), and the OATP-dependent transport activity was determined by comparing the data to those obtained from mock transfected cells. In all experiments, three biological replicates were used and IC_50_ values were calculated by using GraphPad prism software (version 5.01, GraphPad, La Jolla, CA, USA). Due to the high level of specific transporter expression, background transport was very low in these cells (see reference [47]).

## 3. Results

### 3.1. Interaction of Anti-COVID-19 Drug Candidates with ABCB1/MDR1/Pgp

**Figure 1:**
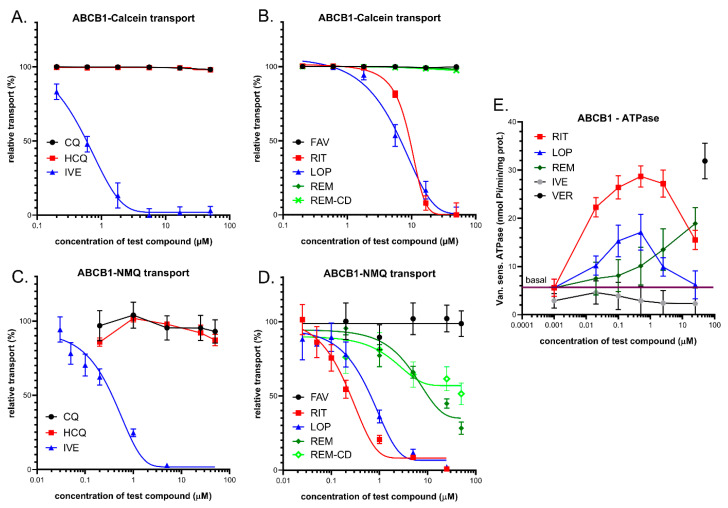
(**A**,**B**) Inhibition of ABCB1-mediated Calcein-AM (CaAM) extrusion in intact ABCB1 expressing PLB-985 cells. (**A**) Effects of ivermectin (IVE), chloroquine (CQ), and hydroxychloroquine (HCQ). (**B**) Effects of lopinavir (LOP), ritonavir (RIT), favipiravir (FAV), remdesivir (REM), and cyclodextrin formulated remdesivir (REM-CD). (**C**,**D**) Inhibition of ABCB1-mediated N-methyl quinidine (NMQ) transport in the vesicular transport assay. (**E**) ABCB1-ATPase activity in isolated Sf9 membrane vesicles. Effects of ivermectin, lopinavir, ritonavir, and remdesivir. As a reference substrate, verapamil (VER) was used. The basal line represents the ATPase activity level without the addition of any drug. Data on the graphs show the average of at least three independent experiments, +/− SD or SEM (**E**) values.

In these experiments, we applied several independent in vitro methods to explore the drug interactions with this transporter. We measured ABCB1-related fluorescent substrate (Calcein-AM) transport inhibition in intact model cells, inhibition of the vesicular transport of an established ABCB1 substrate N-methyl quinidine (NMQ) in mammalian cell membrane vesicles, and drug-dependent ABCB1-ATPase activity in isolated insect (Sf9) cell membranes (see Figure 1). In all cases, previously well-characterized cells or membranes with highly expressed ABCB1 levels were applied, so the data can be compared to numerous similar studies in the relevant scientific literature.

#### 3.1.1. Transport Assays in Intact Human PLB-985/ABCB1 Cells

ABCB1 is a high-affinity active efflux transporter of the viability dye Calcein-AM (CaAM). The cellular uptake of the non-fluorescent CaAM is strongly inhibited by the extrusion of this compound by the ABCB1 transporter, so the cellular fluorescence of the intracellularly formed free Calcein is strongly reduced in cells expressing ABCB1 [53]. Here, we applied this widely used cellular assay (see https://www.solvobiotech.com/services/categories/dye-efflux-assays) to characterize drug interactions of the applied compounds in intact PLB-985 cells, expressing high levels of the ABCB1 protein. Maximum inhibition of the transporter was achieved by 0.25 µM tariquidar (TQ), which is a third-generation specific inhibitor of ABCB1. ABCB1 inhibition by the test compounds was estimated by their relative (%) inhibition compared to full inhibition by TQ. As shown in Figure 1A,B, ivermectin caused a strong inhibition of the ABCB1-dependent CaAM extrusion at low micromolar concentrations (estimated IC_50_ of 0.6 µM), while chloroquine and hydroxychloroquine had practically no effect on this ABCB1-dependent transport. Figure 1B documents the effects of lopinavir, ritonavir, favipiravir, and remdesivir on the CaAM extrusion by ABCB1. Favipiravir, remdesivir, and its cyclodextrin complex did not significantly inhibit the transport activity of ABCB1. In contrast, lopinavir and ritonavir caused a strong inhibition of the ABCB1-dependent CaAM extrusion at low micromolar concentrations (estimated IC_50_ values of 6.3 and 8.4 µM, respectively).

#### 3.1.2. Vesicular Transport Studies in HEK/ABCB1 Membrane Vesicles

In these experiments, we studied the effects of the potential anti-COVID-19 compounds in a vesicular transport assay by using inverted membrane vesicles prepared from HEK-293 cells expressing high levels of the ABCB1 transporter. In the inverted membrane vesicle measurements, labeled substrates and the investigated compounds were both applied on the cytoplasmic side of the membrane, which excludes most of the complex intracellular drug interactions. The modulation of the ATP-dependent vesicular uptake of a specific ABCB1 probe substrate directly reveals drug interactions with the transporter. In the present work, we measured the uptake of 3H-N-methyl-quinidine (NMQ), which is a labeled low-permeability amphipathic substrate of the human ABCB1/MDR1/Pgp [54,55]. Transport inhibition (%) of this labeled substrate by the test compounds was calculated by setting probe substrate transport in the absence of test compounds as 100%. As shown in Figure 1C, in ABCB1 expressing membrane vesicles, ivermectin caused a strong inhibition of the ATP-dependent NMQ uptake at low micromolar concentrations (estimated IC_50_ of 0.3 µM), while chloroquine and hydroxychloroquine had practically no effect on this transport activity. Figure 1D shows the effects of lopinavir, ritonavir, favipiravir, and remdesivir on the transport activity of ABCB1. As documented, lopinavir and ritonavir are strong inhibitors of this transport (estimated IC_50_ values are 0.6 and 0.3 µM, respectively), remdesivir and remdesivir-SBECD are weak inhibitors of ABCB1 (IC_50_ above 20 µM), and favipiravir had no inhibitory effect.

#### 3.1.3. ABCB1-ATPase Activity Measurements in Sf9 Membranes

Drug-stimulated ATPase activity of the ABCB1 transporter is a well-documented functional assay employed to estimate the substrate or inhibitory features of various drugs. ATP-dependent substrate transport is reflected in the activation of this specific ATPase activity in most cases, and in many cases, drug-stimulated ABCB1-ATPase has been shown to correlate with the substrate affinity. In this assay, most transported substrates show a biphasic curve: Low concentrations stimulate, while higher concentrations inhibit, the ATPase activity [46]. In these experiments, we measured the vanadate-sensitive (ABC transporter-related) ABCB1-ATPase activity in membrane vesicles isolated from ABCB1 overexpressing Sf9 cells [46,49]. Sf9 cells exhibit low intrinsic membrane ATPase activity, so both the baseline and the drug-stimulated ATPase activity of the ABCB1 protein can be measured. High-level stimulation of this ATPase activity can be achieved by 50 µM of verapamil—a transported substrate of ABCB1—serving as a positive control in this assay. As shown in Figure 1E, several tested compounds significantly increased the ABCB1-ATPase activity in this assay. Low micromolar concentrations of ritonavir (EC_50_ less than 0.1 µM) stimulated the ATPase activity up to the level of verapamil activation, and both lopinavir (EC_50_ about 0.05 µM) and remdesivir (EC_50_ about 10 µM) showed significant ABCB1-ATPase stimulation. Ritonavir and lopinavir exhibited maximal stimulation at around 0.5 µM, whereas remdesivir only increased the ATPase activity at 10–50 µM. At higher concentrations, ritonavir and lopinavir displayed inhibitory effects. Ivermectin only showed ABCB1-ATPase inhibition.

### 3.2. Interaction of Anti-COVID-19 Drug Candidates with ABCC1/MRP1

#### 3.2.1. Transport Assay in Intact Human Cells—HL60/ABCC1 Cells

**Figure 2:**
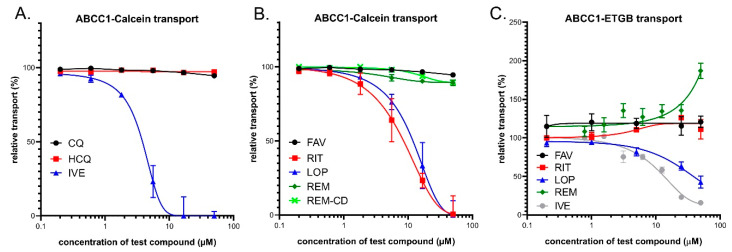
(**A**,**B**) Inhibition of ABCC1-mediated CaAM extrusion in intact HL60 cells. Panel C: Inhibition of ABCC1-mediated vesicular uptake of ^3^H-estradiol-17b-glucuronide (ETGB). Panel (**A**) Effects of ivermectin (IVE), chloroquine (CQ), and hydroxychloroquine (HCQ). (**B**) Effects of lopinavir (LOP), ritonavir (RIT), favipiravir (FAV), remdesivir (REM), and SEBCD-remdesivir (REM-CD). (**C**) Effects of ivermectin (IVE), lopinavir (LOP), ritonavir (RIT), favipiravir (FAV), and remdesivir (REM).

ABCC1, similarly to ABCB1, is a high-affinity active efflux transporter for Calcein-AM and the cellular fluorescence of free Calcein is also reduced in cells expressing ABCC1 [56]. Therefore, we applied this assay to characterize the test drug interactions in intact HL60 cells expressing high levels of the ABCC1 protein. Maximum inhibition of the transporter was achieved by 10 µM of indomethacin (IM), which is a strong inhibitor of ABCC1. ABCC1 inhibition by the test compounds was estimated by their relative (%) inhibition compared to full inhibition by IM. As shown in Figure 2A, ivermectin caused a strong inhibition of the ABCC1-dependent CaAM extrusion at low micromolar concentrations (estimated IC_50_ of 3.3 µM), while chloroquine and hydroxychloroquine had practically no effect. As documented in Figure 2B, lopinavir and ritonavir had strong inhibitory effects, with estimated IC_50_ values of 10.7 and 7.7 µM, respectively. Favipiravir, remdesivir, and its cyclodextrin complex did not significantly inhibit the cellular transport activity of ABCC1 (a slight inhibition by higher concentrations of remdesivir was observed).

#### 3.2.2. Vesicular Transport Studies in Sf9/ABCC1 Membrane Vesicles

In this study, we measured the effects of the test compounds in a vesicular transport assay by using inverted membrane vesicles prepared from Sf9 cells expressing high levels of the ABCC1 transporter. We measured the uptake of ^3^H-estradiol-17b-glucuronide (ETGB), which is a labeled substrate of the human ABCC1/MRP1 transporter [57]. Relative inhibition (%) was calculated by setting probe substrate transport in the absence of test compounds as 100%. As shown in Figure 2C, in ABCC1 expressing membrane vesicles, lopinavir caused an inhibition of the ATP-dependent ETGB uptake in a dose-dependent manner, with a maximum inhibition of 60% at the highest applied concentration. Ivermectin inhibited the ABCC1-mediated ETBG accumulation, with a maximum inhibition of 80% at a 25 μM concentration. Ritonavir, favipiravir, chloroquine, and hydroxychloroquine had practically no effect on this transport activity (CQ and HCQ not shown), while, interestingly, remdesivir significantly stimulated the vesicular ETGB transport activity of ABCC1, although only at above 25 μM.

### 3.3. Interaction of Anti-COVID-19 Drug Candidates with ABCG2

#### 3.3.1. Transport Measurements in Intact Human Cells—PLB/ABCG2 and HeLa/ABCG2

**Figure 3:**
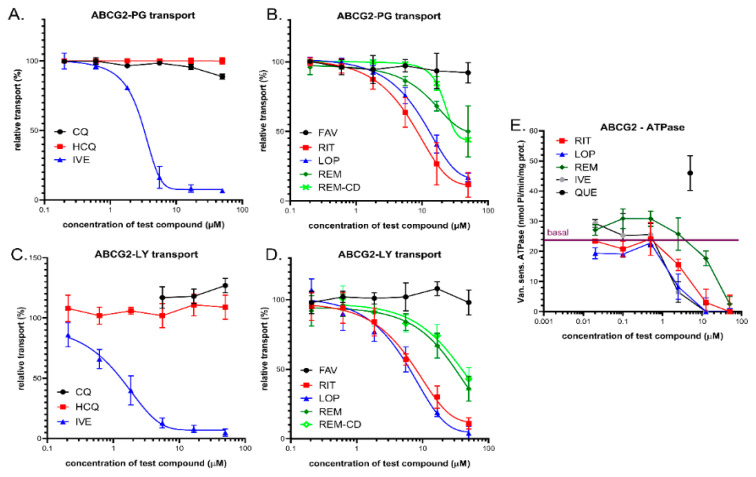
(**A**,**B**) Inhibition of ABCG2-mediated PhenGreen (PG)-AM extrusion in intact PLB-985 cells. Panel A: Effects of ivermectin (IVE), chloroquine (CQ), and hydroxychloroquine (HCQ). Panel B: Effects of lopinavir (LOP), ritonavir (RIT), favipiravir (FAV), remdesivir (REM), and SEBCD-remdesivir (REM-CD). (**C**,**D**) Inhibition of ABCG2-mediated lucifer yellow (LY) transport in the vesicular transport assay. (**E**) ABCG2-ATPase activity in isolated Sf9 membrane vesicles. Effects of ivermectin, lopinavir, ritonavir, and remdesivir. Maximum ATPase stimulation was obtained by 5 µM quercetin. Data on the graphs show the average of at least three independent experiments, +/− SD (**A**,**B**) or SEM (**C**–**E**) values.

The ABCG2 protein does not transport Calcein AM, so this assay cannot be used for assaying ABCG2 activity in intact cells. In contrast, several fluorescent dyes, including Hoechst 33342 (Hst), DyeCycle violet (DCV), and PhenGreen-SK diacetate (PG-DA), are actively extruded by the ABCG2 transporter [44,58,59,60,61,62,63], providing an opportunity for fluorescence-based cellular assays. Here, we used both PhenGreen-SK diacetate and Hst dye for these measurements. The PhenGreen (PG) assay is a recently patented method applied for ensuring an efficient ABCG2 transport measurement, as the non-fluorescent PhenGreen diacetate (PG-DA) is actively extruded by ABCG2 and the cellular fluorescence is correlated with ABCG2 transport activity [44]. Therefore, we applied this assay to characterize the test drug interactions in intact PLB-985 cells expressing high levels of the ABCG2 protein. Maximum inhibition of the transporter was achieved by 2.5 µM of Ko143, which is a high-affinity specific inhibitor of ABCG2. ABCG2 inhibition by the test compounds was estimated by their relative (%) inhibition compared to full inhibition by Ko143. As shown in Figure 3A,B, ivermectin caused a strong inhibition of the ABCG2-dependent PG-DA extrusion at micromolar concentrations (estimated IC_50_ of 3.1 µM). Lopinavir and ritonavir also had strong inhibitory effects, with estimated IC_50_ values of 13.1 and 8.3 µM, respectively. Favipiravir, chloroquine, and hydroxychloroquine had practically no effect on this ABCG2-dependent transport. Remdesivir and its complex are relatively weak inhibitors of the ABCG2-dependent PG-DA extrusion at micromolar concentrations (estimated IC_50_ values are greater than 40–50 µM).

#### 3.3.2. Vesicular Transport Studies in HEK/ABCG2 Membrane Vesicles

The effects of the test compounds on ABCG2 activity were also measured in a vesicular transport assay by using inverted membrane vesicles prepared from HEK-293 cells expressing high levels of the ABCG2 transporter. The ATP-dependent uptake of lucifer yellow (LY), which is a substrate of the human ABCG2/BCRP transporter [64], was measured, and inhibition of this fluorescent substrate uptake by the test compounds was compared to a full inhibition achieved by 1 µM of Ko143. As shown in Figure 3C,D, in ABCG2 containing HEK-293 cell membrane vesicles, ivermectin caused a strong inhibition of the ATP-dependent LY uptake at low micromolar concentrations (estimated IC_50_ of 1.1 µM), while chloroquine and hydroxychloroquine had practically no effect. Lopinavir, ritonavir, and remdesivir, as well as remdesivir-cyclodextrin, inhibited the transport activity of ABCG2, with estimated IC_50_ values of 4.2, 7.5, and more than 20 µM, respectively.

#### 3.3.3. ABCG2-ATPase Activity Measurements in Sf9 Membranes

Drug-stimulated ATPase activity of the ABCG2 transporter was also measured in the present study. This functional assay may be used to estimate the substrate/inhibitory features of various drugs (see 1.E, and [50]). Here, we measured the vanadate-sensitive ABCG2-ATPase activity in membrane vesicles isolated from ABCG2 overexpressing Sf9 cells [50], and the maximum stimulation of this ABCG2-ATPase activity was achieved by the transported substrate—5 µM quercetin—serving as a positive control in this assay. As shown in Figure 3E, ivermectin, ritonavir, and lopinavir at low micromolar concentrations, and remdesivir at higher concentrations (around 20–50 micromoles), significantly inhibited the baseline ABCG2-ATPase activity. None of the tested compounds displayed significant ABCG2-ATPase stimulation effects. Therefore, the assay was not informative regarding their potential transported substrate nature.

#### 3.3.4. Effect of the Q141K-ABCG2 Polymorphism on the Inhibitory Potential of the Test Drugs

**Figure 4:**
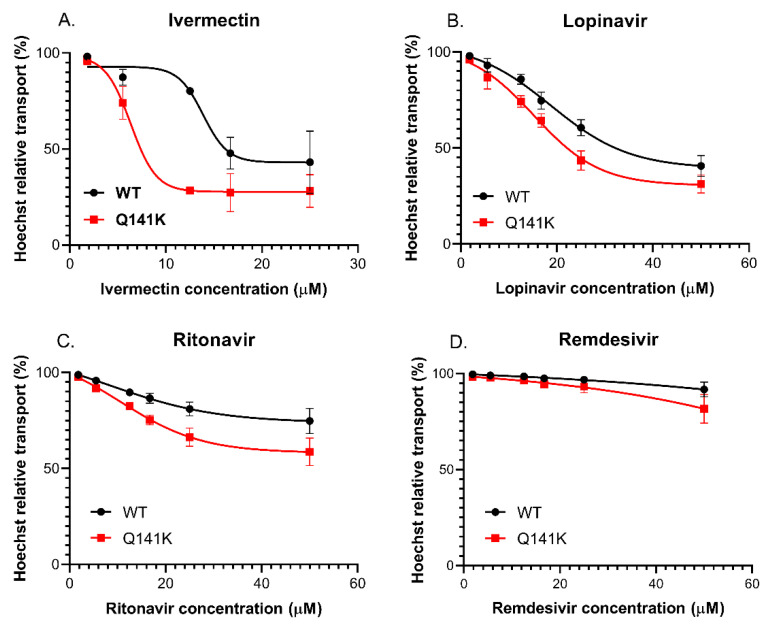
Hoechst 33342 dye extrusion in HeLa cells stably expressing the WT and Q141K variants of ABCG2. Different concentrations of ivermectin (**A**), lopinavir (**B**), ritonavir (**C**), and remdesivir (**D**) were used to determine their effect on ABCG2 function. Data on the graphs show the average of three independent experiments, +/− SD values.

The very frequent (present in 12–35% in various populations) Q141K-ABCG2 polymorphic variant has been reported to have lower membrane expression levels and reduced transport activity in various assay systems [65]. Since ivermectin, lopinavir, ritonavir, and remdesivir resulted in well-measurable inhibition of the ABCG2-dependent PG dye extrusion activity (see above), we performed similar studies in HeLa cells stably expressing either the wild-type ABCG2 or the Q141K-ABCG2 variant [45]. In this case, we measured the extrusion of the Hoechst 33342 dye, which is another transported substrate of ABCG2, and once again used the specific inhibitor Ko143 to achieve full inhibition of the transporter. As shown in Figure 4, ivermectin (A), ritonavir (B), and lopinavir (C) also inhibited Hst dye extrusion by ABCG2 in the HeLa cells, and remdesivir (D) had a slight effect at relatively high concentrations. An important finding was that Hst dye extrusion in HeLa cells expressing the Q141K-ABCG2 variant showed a higher sensitivity to all drug inhibition.

### 3.4. Interaction of Anti-COVID-19 Candidates with OATP1A2 and OATP2B1 Transporters

**Figure 5:**
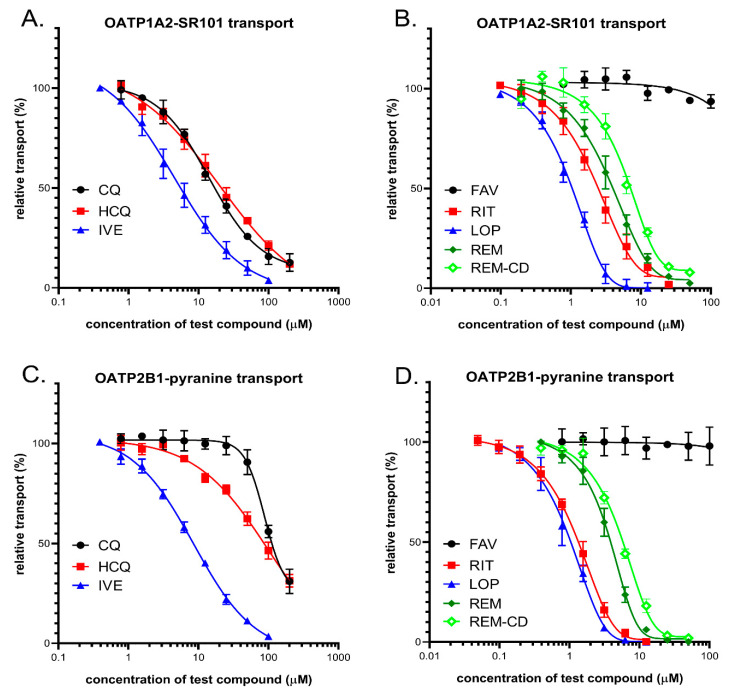
(**A**,**B**) Inhibition of OATP1A2-mediated sulforhodamine101 (SR101) uptake by potential antiviral compounds. Uptake of 0.5 μM SR101 was measured in A431-OATP1A2 cells seeded on 96-well plates for 10 min in the presence of increasing concentrations of ivermectin (IVE), chloroquine (CQ), hydroxychloroquine (HCQ), favipiravir(FAV), lopinavir (LOP), ritonavir (RIT), remdesivir (REM), and SEBCD-remdesivir. (**C**,**D**) Inhibition of OATP2B1-mediated pyranine uptake by different antiviral compounds. Uptake of 20 μM pyranine was measured in A431-OATP2B1 cells for 15 min in increasing concentrations of the tested compounds. In all cases, averages were obtained based on at least three biological replicates. +/− SD values are shown.

In order to explore potential interactions between the potential anti-COVID-19 drugs and OATP1A2 or OATP2B1, the compounds were investigated in a fluorescence-based cellular transport assay recently developed by our laboratory [47,51]. The uptake of pyranine or sulforhodamine 101 as test substrates was measured in A431-OATP1A2 or A431-OATP2B1 cells, and mock transfected A431 were used as the negative control. As shown by Figure 5, with the exception of favipiravir, all of the compounds examined inhibited OATP1A2 function. Based on the IC_50_ values, the antivirals lopinavir, ritonavir, remdesivir, and the anti-parasitic ivermectin exhibited similar affinities towards OATP1A2. On the other hand, although still effective, chloroquine and hydroxychloroquine were 3–10-fold lower affinity inhibitors (see Table 2). Interestingly, cyclodextrin resulted in a slightly decreased inhibitory potential of remdesivir on OATP1A2 function (Figure 5, and Table 2). In the case of OATP2B1, a similar inhibitory potency was observed for the antiviral compounds, as in the case of OATP1A2. Lopinavir and ritonavir were the highest affinity inhibitors, with IC_50_ values of 1.0 and 1.4 μM, respectively, and remdesivir, the remdesivir-cyclodextrin complex, and ivermectin had lower inhibitory effects, with IC_50_ values of 3.8, 5.6, and 8.6 μM, respectively. Chloroquine and hydroxychloroquine exerted only modest inhibition of OATP2B1 activity, and favipiravir showed no interaction with this transporter.

## 4. Discussion

Multispecific drug and xenobiotic transporters play a major role in the pharmacokinetics of numerous pharmacological agents, and any new drug candidates have to be tested for interactions with the key transporters in this regard [66,67]. However, several drugs rapidly repurposed in the past months for potential anti-COVID-19 activity may not have been analyzed in detail for these interactions. This lack of information is making their clinical use, especially in combination with other pharmacological agents, potentially dangerous for patients. Since SARS-CoV-2 virus infection causes severe clinical symptoms, especially in elderly patients and/or in those with existing co-morbidities, the ADME-Tox properties and the potentially harmful drug–drug interactions of the repurposed drugs may have major relevance in these cases. In addition, there are no current methods for correctly estimating the transporter–drug interactions by in silico approaches, and only detailed in vitro studies may answer these questions. Therefore, in the present study, we examined the interactions of the repurposed drug candidates with the key multispecific drug transporters, with the hope that our results help clinical applications in COVID-19 treatment. We have used a wide range of assay systems and transported substrates to provide comparative aspects for the transporter–drug interactions.

ABC transporters play a key role in the pharmacokinetics of numerous pharmacological agents, and they—especially ABCB1 and ABCG2—should be tested in early phases of drug development [66]. As shown in the results section and in the summary in Table 2, regarding the three ABC multispecific transporters examined, we found that chloroquine, hydroxychloroquine, and favipiravir displayed practically no potentially relevant interaction with any of these transporters. In contrast, ivermectin was found to exert a strong inhibitory effect in the case of the ABCB1, ABCC1, and ABCG2 transporter. Some of these interactions have already been explored; in particular, ivermectin inhibition of the human and animal Mdr1/Pgp/ABCB1 has been studied in detail [8,9,68]. As we show here, in all kinds of assays, a strong inhibition of both ABCC1 and ABCG2 transport activity was also observed by ivermectin, emphasizing the potentially dangerous effects of this compound at higher doses or with an impaired transporter function. The antiviral protease inhibitors lopinavir and ritonavir had a significant inhibitory effect on the three ABC transporters examined here, and a strong inhibition was observed for ABCB1/Pgp (Table 2). However, ABCC1 and ABCG2 were also inhibited by these compounds in potentially relevant concentrations. As suggested by the relevant FDA information (https://www.accessdata.fda.gov/drugsatfda_docs/label/2007/021226s018lbl.pdf, https://www.ema.europa.eu/en/documents/scientific-discussion/kaletra-epar-scientific-discussion_en.pdf), lopinavir in patients may reach plasma concentrations of about 13 μM, while ritonavir may peak at about 1 μM. Therefore, our in vitro data indicate that drug–drug interactions should be considered. Remdesivir was found to be a relatively weak inhibitor of all three ABC transporters, although the ABCB1/Pgp inhibition with an IC_50_ of about 20 μM, which was observed in the vesicular transport assay, may be relevant under certain treatment conditions (reported peak plasma concentrations of remdesivir of about 5 μM). Interestingly, remdesivir significantly stimulated the vesicular transport of a test substrate ETGB by the ABCC1 transporter, so an allosteric effect of remdesivir on this transporter should be considered.

The experiments performed in intact cells expressing the polymorphic ABCG2 transporter variant Q141K, with impaired membrane localization and transport activity, suggest that, with lower transporter functions, some of the weak inhibitors may have a clinically important effect. In this case, both lopinavir and ritonavir exerted significantly stronger inhibition of this drug transporter variant. Considering that the allele encoding the ABCG2-Q141K variant (rs2231142) has an incidence of about 30% in the Asian population, this information is especially important for clinical interventions in these countries. In our studies, we also examined the membrane ATPase activity of the ABCB1 and ABCG2 transporters in isolated membranes. These assays may help to decipher the substrate vs. inhibitor nature of the test compounds. Although these results only provide a tentative answer in this regard, we found that the ABCB1-ATPase activity is significantly stimulated by low concentrations of lopinavir, ritonavir, and remdesivir, indicating that these drugs may be transported substrates. We observed no such stimulation in the case of the ABCG2 transporter in terms of these drugs. In addition to the ABC multidrug transporters, the role of drug-transporting OATPs in pharmacokinetics and drug–drug or food–drug interactions is increasingly being recognized [66]. While OATPs 1B1 and 1B3 are liver-specific proteins, OATP1A2 and OATP2B1 are highly expressed in the endothelial cells of the BBB. Therefore, these transporters are key modulators of the entry of their drug substrates into the central nervous system [41,69,70]. In addition, OATP2B1 also influences the absorption of its substrates from the intestine [40]. In the current study, we investigated the interactions between OATP1A2 and OATP2B1 and drugs repurposed to treat COVID-19 disease. We found high-affinity interactions between the antiviral protease inhibitors lopinavir and ritonavir with both OATP1A2 and OATP2B1. These interactions have already been documented [71,72,73,74]. Moreover, lopinavir and ritonavir are also inhibitors of OATP1B1 and OATP1B3, which are the key transporters in the liver [71]. The IC_50_ values obtained in our study are in harmony with those observed by Tupova et al. [72] and Kis et al. [73]. Moreover, a previous study [74] showed that lopinavir is transported by OATP1A2, so OATP1A2 probably affects lopinavir absorption and blood to brain entry. Ivermectin, as reported previously [75], also inhibited OATP2B1, although in our experiments, with a higher affinity than that found by Karlgren et al. (20 µM, 39%—this may be explained by the different test substrates used). However, here, we document, for the first time, that ivermectin inhibits OATP1A2 function. Our experiments confirmed the relatively weak interactions between chloroquine and hydroxychloroquine with OATP1A2 and OATP2B1, and the observed IC_50_ values are in harmony with those found by others [76,77]. We observed no interaction of favipiravir with the two OATPs examined here. There are no data available for the interaction of remdesivir–currently a major anti-COVID-19 drug candidate–with OATP1A2 and OATP2B1. Based on our data, showing a high-affinity interaction of remdesivir with both transporters, and the relatively high plasma concentrations of this drug (peak values in 3–5 μM [78,79]), remdesivir may significantly interfere with the pharmacokinetics of drug substrates of OATP1A2 and OATP2B1. Our current methods do not distinguish between transported and non-transported inhibitors, so further investigations are needed to decipher whether OATP1A2 and OATP2B1 can mediate the uptake of remdesivir. From the currently developed anti-COVID-19 drugs tested in our study, favipiravir is the most promising candidate for avoiding unexpected drug–drug interactions. Favipiravir, similar to what was found for the multispecific ABC transporters located in the tissue barriers (see above), did not inhibit the function of the investigated OATPs. Therefore, this compound is not expected to influence OATP-mediated drug pharmacokinetics. As a summary, our current data, summarized in Table 2, provide a detailed in vitro quantitative analysis on the interaction of the anti-COVID-19 agents with the key human multispecific drug transporters. These in vitro assays, which should be followed by careful clinical pharmacokinetic studies, may provide a strong warning against the compassionate use of some of these agents, especially when other relevant drugs are also applied to the patient, or in cases of endogenously impaired transporter activity.

## Figures and Tables

**Table 1:** Mechanism of action of the potential anti-COVID-19 drugs examined in this study.

Potential Anti-COVID-19 Compounds	Mechanism of Action
chloroquine	Antimalarial—endosomal pH increase
hydroxychloroquine	Antimalarial—endosomal pH increase
ivermectin	Antiparasitic—glutamate-gated chloride channel and a GABA receptor inhibitor
lopinavir	(HIV) protease inhibitor
ritonavir	(HIV) protease inhibitor
remdesivir	Viral RNA-polymerase inhibitor
favipiravir	Viral RNA-polymerase inhibitor

**Table 2:** Summary of the transporter inhibition properties of the drugs examined. Approximate IC_50_ (μM) values were determined by nonlinear regression analysis of the data shown in the results section, using GraphPad prism software (version 5.01, GraphPad, La Jolla, CA, USA).

Estimated Transporter Inhibition—IC_50_ (µM)								
	ABCB1		ABCC1		ABCG2		OATP Cellular Assays	
Potential anti-COVID-19 compounds	cellular assay	vesicular assay	cellular assay	vesicular assay	cellular assay	vesicular assay	OATP1A2	OATP2B1
chloroquine	-	-	-	-	-	-	17.0	119
hydroxychloroquine	-	-	-	-	-	-	18.9	84
ivermectin	0.6	0.3	3.3	13.3	3.1	1.1	5.2	8.6
lopinavir	6.3	0.6	10.7	10	13.1	4.2	1.5	1.0
ritonavir	8.4	0.3	7.7	-	8.3	7.5	2.3	1.4
remdesivir	-	>20	-	-*	>50	>50	3.8	3.8
remdesivir-SBECD	-	>20	-	NA	>50	>50	6.1	5.6
favipiravir	-	-	-	-	-	-	-	-

* Stimulation of substrate transport.

## Data Availability

The data presented in this study are available on request from the corresponding author at the Researchgate website (https://www.researchgate.net/profile/Csilla_Ozvegy-Laczka, or https://www.researchgate.net/publication/347138768_Interactions_of_anti-COVID-19_drug_candidates_with_multispecific_ABC_and_OATP_drug_transporters). The data are not publicly available due to a company participation.

## References

[1] Savarino A., Boelaert J.R., Cassone A., Majori G., Cauda R. (2003). Effects of chloroquine on viral infections: An old drug against today’s diseases?. Lancet Infect. Dis..

[2] Singh H., Chauhan P., Kakkar A.K. (2020). Hydroxychloroquine for the treatment and prophylaxis of COVID-19: The journey so far and the road ahead. Eur. J. Pharmacol..

[3] Vincent M.J., Bergeron E., Benjannet S., Erickson B.R., Rollin P.E., Ksiazek T.G., Seidah N.G., Nichol S.T. (2005). Chloroquine is a potent inhibitor of SARS coronavirus infection and spread. Virol. J..

[4] Liu J., Cao R., Xu M., Wang X., Zhang H., Hu H., Li Y., Hu Z., Zhong W., Wang M. (2020). Hydroxychloroquine, a less toxic derivative of chloroquine, is effective in inhibiting SARS-CoV-2 infection in vitro. Cell Discov..

[5] Wang M., Cao R., Zhang L., Yang X., Liu J., Xu M., Shi Z., Hu Z., Zhong W., Xiao G. (2020). Remdesivir and chloroquine effectively inhibit the recently emerged novel coronavirus (2019-nCoV) in vitro. Cell Res..

[6] Simpson T.F., Kovacs R.J., Stecker E.C. Cardiology Magazine.

[7] Roden D.M., Harrington R.A., Poppas A., Russo A.M. (2020). Considerations for Drug Interactions on QTc in Exploratory COVID-19 Treatment. Circulation.

[8] Schinkel A.H., Smit J.J., van Tellingen O., Beijnen J.H., Wagenaar E., van Deemter L., Mol C.A., van der Valk M.A., Robanus-Maandag E.C., te Riele H.P. (1994). Disruption of the mouse mdr1a P-glycoprotein gene leads to a deficiency in the blood-brain barrier and to increased sensitivity to drugs. Cell.

[9] Mealey K.L., Bentjen S.A., Gay J.M., Cantor G.H. (2001). Ivermectin sensitivity in collies is associated with a deletion mutation of the mdr1 gene. Pharmacogenetics.

[10] Lespine A., Dupuy J., Orlowski S., Nagy T., Glavinas H., Krajcsi P., Alvinerie M. (2006). Interaction of ivermectin with multidrug resistance proteins (MRP1, 2 and 3). Chem. Biol. Interact..

[11] Pouliot J.F., L’Heureux F., Liu Z., Prichard R.K., Georges E. (1997). Reversal of P-glycoprotein-associated multidrug resistance by ivermectin. Biochem. Pharmacol..

[12] Didier A., Loor F. (1996). The abamectin derivative ivermectin is a potent P-glycoprotein inhibitor. Anticancer. Drugs.

[13] Wagstaff K., Sivakumaran H., Heaton S., Harrich D., Jans D. (2012). Ivermectin is a specific inhibitor of importin alpha/beta-mediated nuclear import able to inhibit replication of HIV-1 and Dengue virus. Biochem. J..

[14] Lundberg L., Pinkham C., Baer A., Amaya M., Narayanan A., Wagstaff K.M., Jans D.A., Kehn-Hall K. (2013). Nuclear import and export inhibitors alter capsid protein distribution in mammalian cells and reduce Venezuelan Equine Encephalitis Virus replication. Antivir. Res..

[15] Yang S.N.Y., Atkinson S.C., Wang C., Lee A., Bogoyevitch M.A., Borg N.A., Jans D.A. (2020). The broad spectrum antiviral ivermectin targets the host nuclear transport importin α/β1 heterodimer. Antivir. Res..

[16] Bray M., Rayner C., Noël F., Jans D., Wagstaff K. (2020). Ivermectin and COVID-19: A report in Antiviral Research, widespread interest, an FDA warning, two letters to the editor and the authors’ responses. Antivir. Res..

[17] Caly L., Druce J.D., Catton M.G., Jans D.A., Wagstaff K.M. (2020). The FDA-approved drug ivermectin inhibits the replication of SARS-CoV-2 in vitro. Antivir. Res..

[18] Chaccour C., Hammann F., Ramón-García S., Rabinovich N.R. (2020). Ivermectin and COVID-19: Keeping Rigor in Times of Urgency. Am. J. Trop. Med. Hyg..

[19] Uzunova K., Filipova E., Pavlova V., Vekov T. (2020). Insights into antiviral mechanisms of remdesivir, lopinavir/ritonavir and chloroquine/hydroxychloroquine affecting the new SARS-CoV-2. Biomed. Pharmacother..

[20] Arshad U., Pertinez H., Box H., Tatham L., Rajoli R.K.R., Curley P., Neary M., Sharp J., Liptrott N.J., Valentijn A. (2020). Prioritization of Anti-SARS-Cov-2 Drug Repurposing Opportunities Based on Plasma and Target Site Concentrations Derived from their Established Human Pharmacokinetics. Clin. Pharmacol. Ther..

[21] Weiss J., Rose J., Storch C.H., Ketabi-Kiyanvash N., Sauer A., Haefeli W.E., Efferth T. (2007). Modulation of human BCRP (ABCG2) activity by anti-HIV drugs. J. Antimicrob. Chemother..

[22] Martinec O., Huliciak M., Staud F., Cecka F., Vokral I., Cerveny L. (2019). Anti-HIV and Anti-Hepatitis C Virus Drugs Inhibit P-Glycoprotein Efflux Activity in Caco-2 Cells and Precision-Cut Rat and Human Intestinal Slices. Antimicrob. Agents Chemother..

[23] Corona G., Vaccher E., Sandron S., Sartor I., Tirelli U., Innocenti F., Toffoli G. (2008). Lopinavir-ritonavir dramatically affects the pharmacokinetics of irinotecan in HIV patients with Kaposi’s sarcoma. Clin. Pharmacol. Ther..

[24] Agarwal S., Pal D., Mitra A.K. (2007). Both P-gp and MRP2 mediate transport of Lopinavir, a protease inhibitor. Int. J. Pharm..

[25] Janneh O., Jones E., Chandler B., Owen A., Khoo S.H. (2007). Inhibition of P-glycoprotein and multidrug resistance-associated proteins modulates the intracellular concentration of lopinavir in cultured CD4 T cells and primary human lymphocytes. J. Antimicrob. Chemother..

[26] Gupta A., Zhang Y., Unadkat J.D., Mao Q. (2004). HIV protease inhibitors are inhibitors but not substrates of the human breast cancer resistance protein (BCRP/ABCG2). J. Pharmacol. Exp. Ther..

[27] Bierman W.F.W., Scheffer G.L., Schoonderwoerd A., Jansen G., van Agtmael M.A., Danner S.A., Scheper R.J. (2010). Protease inhibitors atazanavir, lopinavir and ritonavir are potent blockers, but poor substrates, of ABC transporters in a broad panel of ABC transporter-overexpressing cell lines. J. Antimicrob. Chemother..

[28] Yoon J.-J., Toots M., Lee S., Lee M.-E., Ludeke B., Luczo J.M., Ganti K., Cox R.M., Sticher Z.M., Edpuganti V. (2018). Orally Efficacious Broad-Spectrum Ribonucleoside Analog Inhibitor of Influenza and Respiratory Syncytial Viruses. Antimicrob. Agents Chemother..

[29] Furuta Y., Komeno T., Nakamura T. (2017). Favipiravir (T-705), a broad spectrum inhibitor of viral RNA polymerase. Proc. Jpn. Acad. Ser. B Phys. Biol. Sci..

[30] Yang K. (2020). What Do We Know About Remdesivir Drug Interactions?. Clin. Transl. Sci..

[31] Szakács G., Váradi A., Ozvegy-Laczka C., Sarkadi B. (2008). The role of ABC transporters in drug absorption, distribution, metabolism, excretion and toxicity (ADME-Tox). Drug Discov. Today.

[32] Takada T., Ichida K., Matsuo H., Nakayama A., Murakami K., Yamanashi Y., Kasuga H., Shinomiya N., Suzuki H. (2014). ABCG2 dysfunction increases serum uric acid by decreased intestinal urate excretion. Nucleosides Nucleotides Nucleic Acids.

[33] Sarkadi B., Homolya L., Szakács G., Váradi A. (2006). Human Multidrug Resistance ABCB and ABCG Transporters: Participation in a Chemoimmunity Defense System. Physiol. Rev..

[34] Dauchy S., Dutheil F., Weaver R.J., Chassoux F., Daumas-Duport C., Couraud P.-O., Scherrmann J.-M., De Waziers I., Declèves X. (2008). ABC transporters, cytochromes P450 and their main transcription factors: Expression at the human blood-brain barrier. J. Neurochem..

[35] Kamiie J., Ohtsuki S., Iwase R., Ohmine K., Katsukura Y., Yanai K., Sekine Y., Uchida Y., Ito S., Terasaki T. (2008). Quantitative atlas of membrane transporter proteins: Development and application of a highly sensitive simultaneous LC/MS/MS method combined with novel in-silico peptide selection criteria. Pharm. Res..

[36] Uchida Y., Ohtsuki S., Katsukura Y., Ikeda C., Suzuki T., Kamiie J., Terasaki T. (2011). Quantitative targeted absolute proteomics of human blood-brain barrier transporters and receptors. J. Neurochem..

[37] Daood M., Tsai C., Ahdab-Barmada M., Watchko J.F. (2008). ABC transporter (P-gp/ABCB1, MRP1/ABCC1, BCRP/ABCG2) expression in the developing human CNS. Neuropediatrics.

[38] Hagenbuch B., Gui C. (2008). Xenobiotic transporters of the human organic anion transporting polypeptides (OATP) family. Xenobiotica.

[39] Hagenbuch B., Stieger B. (2013). The SLCO (former SLC21) superfamily of transporters. Mol. Asp. Med..

[40] Shitara Y., Maeda K., Ikejiri K., Yoshida K., Horie T., Sugiyama Y. (2013). Clinical significance of organic anion transporting polypeptides (OATPs) in drug disposition: Their roles in hepatic clearance and intestinal absorption. Biopharm. Drug Dispos..

[41] Urquhart B.L., Kim R.B. (2009). Blood-brain barrier transporters and response to CNS-active drugs. Eur. J. Clin. Pharmacol..

[42] Yu J., Zhou Z., Tay-Sontheimer J., Levy R.H., Ragueneau-Majlessi I. (2017). Intestinal Drug Interactions Mediated by OATPs: A Systematic Review of Preclinical and Clinical Findings. J. Pharm. Sci..

[43] Kovacsics D., Patik I., Özvegy-Laczka C. (2017). The role of organic anion transporting polypeptides in drug absorption, distribution, excretion and drug-drug interactions. Expert Opin. Drug Metab. Toxicol..

[44] Szabó E., Türk D., Telbisz Á., Kucsma N., Horváth T., Szakács G., Homolya L., Sarkadi B., Várady G. (2018). A new fluorescent dye accumulation assay for parallel measurements of the ABCG2, ABCB1 and ABCC1 multidrug transporter functions. PLoS ONE.

[45] Zámbó B., Mózner O., Bartos Z., Török G., Várady G., Telbisz Á., Homolya L., Orbán T.I., Sarkadi B. (2020). Cellular expression and function of naturally occurring variants of the human ABCG2 multidrug transporter. Cell. Mol. Life Sci..

[46] Sarkadi B., Bauzon D., Huckle W.R., Earp H.S., Berry A., Suchindran H., Price E.M., Olson J.C., Boucher R.C., Scarborough G.A. (1992). Biochemical characterization of the cystic fibrosis transmembrane conductance regulator in normal and cystic fibrosis epithelial cells. J. Biol. Chem..

[47] Patik I., Székely V., Német O., Szepesi Á., Kucsma N., Várady G., Szakács G., Bakos É., Özvegy-Laczka C. (2018). Identification of novel cell-impermeant fluorescent substrates for testing the function and drug interaction of Organic Anion-Transporting Polypeptides, OATP1B1/1B3 and 2B1. Sci. Rep..

[48] Ozvegy C., Litman T., Szakács G., Nagy Z., Bates S., Váradi A., Sarkadi B. (2001). Functional characterization of the human multidrug transporter, ABCG2, expressed in insect cells. Biochem. Biophys. Res. Commun..

[49] Telbisz A., Müller M., Ozvegy-Laczka C., Homolya L., Szente L., Váradi A., Sarkadi B. (2007). Membrane cholesterol selectively modulates the activity of the human ABCG2 multidrug transporter. Biochim. Biophys. Acta.

[50] Ozvegy C., Váradi A., Sarkadi B. (2002). Characterization of drug transport, ATP hydrolysis, and nucleotide trapping by the human ABCG2 multidrug transporter. Modulation of substrate specificity by a point mutation. J. Biol. Chem..

[51] Bakos É., Német O., Patik I., Kucsma N., Várady G., Szakács G., Özvegy-Laczka C. (2020). A novel fluorescence-based functional assay for human OATP1A2 and OATP1C1 identifies interaction between third-generation P-gp inhibitors and OATP1A2. FEBS J..

[52] Székely V., Patik I., Ungvári O., Telbisz Á., Szakács G., Bakos É., Özvegy-Laczka C. (2020). Fluorescent probes for the dual investigation of MRP2 and OATP1B1 function and drug interactions. Eur. J. Pharm. Sci. Off. J. Eur. Fed. Pharm. Sci..

[53] Homolya L., Holló Z., Müller M., Mechetner E.B., Sarkadi B. (1996). A new method for quantitative assessment of P-glycoprotein-related multidrug resistance in tumour cells. Br. J. Cancer.

[54] Hooiveld G.J.E.J., Heegsma J., van Montfoort J.E., Jansen P.L.M., Meijer D.K.F., Müller M. (2002). Stereoselective transport of hydrophilic quaternary drugs by human MDR1 and rat Mdr1b P-glycoproteins. Br. J. Pharmacol..

[55] Herédi-Szabó K., Palm J.E., Andersson T.B., Pál Á., Méhn D., Fekete Z., Beéry E., Jakab K.T., Jani M., Krajcsi P. (2013). A P-gp vesicular transport inhibition assay—optimization and validation for drug-drug interaction testing. Eur. J. Pharm. Sci. Off. J. Eur. Fed. Pharm. Sci..

[56] Holló Z., Homolya L., Hegedûs T., Müller M., Szakács G., Jakab K., Antal F., Sarkadi B. (1998). Parallel functional and immunological detection of human multidrug resistance proteins, P-glycoprotein and MRP1. Anticancer Res..

[57] Slot A.J., Wise D.D., Deeley R.G., Monks T.J., Cole S.P.C. (2008). Modulation of human multidrug resistance protein (MRP) 1 (ABCC1) and MRP2 (ABCC2) transport activities by endogenous and exogenous glutathione-conjugated catechol metabolites. Drug Metab. Dispos..

[58] Strouse J.J., Ivnitski-Steele I., Waller A., Young S.M., Perez D., Evangelisti A.M., Ursu O., Bologa C.G., Carter M.B., Salas V.M. (2013). Fluorescent substrates for flow cytometric evaluation of efflux inhibition in ABCB1, ABCC1, and ABCG2 transporters. Anal. Biochem..

[59] Telford W.G., Bradford J., Godfrey W., Robey R.W., Bates S.E. (2007). Side population analysis using a violet-excited cell-permeable DNA binding dye. Stem Cells.

[60] Boesch M., Reimer D., Rumpold H., Zeimet A.G., Sopper S., Wolf D. (2012). DyeCycle Violet used for side population detection is a substrate of P-glycoprotein. Cytom. A.

[61] Nerada Z., Hegyi Z., Szepesi Á., Tóth S., Hegedüs C., Várady G., Matula Z., Homolya L., Sarkadi B., Telbisz Á. (2016). Application of fluorescent dye substrates for functional characterization of ABC multidrug transporters at a single cell level. Cytom. A.

[62] Zong Y., Zhou S., Fatima S., Sorrentino B.P. (2006). Expression of mouse Abcg2 mRNA during hematopoiesis is regulated by alternative use of multiple leader exons and promoters. J. Biol. Chem..

[63] Zhou S., Schuetz J.D., Bunting K.D., Colapietro A.M., Sampath J., Morris J.J., Lagutina I., Grosveld G.C., Osawa M., Nakauchi H. (2001). The ABC transporter Bcrp1/ABCG2 is expressed in a wide variety of stem cells and is a molecular determinant of the side-population phenotype. Nat. Med..

[64] Sjöstedt N., van den Heuvel J.J.M.W., Koenderink J.B., Kidron H. (2017). Transmembrane Domain Single-Nucleotide Polymorphisms Impair Expression and Transport Activity of ABC Transporter ABCG2. Pharm. Res..

[65] Mózner O., Bartos Z., Zámbó B., Homolya L., Hegedűs T., Sarkadi B. (2019). Cellular Processing of the ABCG2 Transporter-Potential Effects on Gout and Drug Metabolism. Cells.

[66] Giacomini K.M., Balimane P.V., Cho S.K., Eadon M., Edeki T., Hillgren K.M., Huang S.-M., Sugiyama Y., Weitz D., Wen Y. (2013). International Transporter Consortium commentary on clinically important transporter polymorphisms. Clin. Pharmacol. Ther..

[67] Huang S.-M., Zhang L., Giacomini K.M. (2010). The International Transporter Consortium: A collaborative group of scientists from academia, industry, and the FDA. Clin. Pharmacol. Ther..

[68] Geyer J., Gavrilova O., Petzinger E. (2009). Brain penetration of ivermectin and selamectin in mdr1a,b P-glycoprotein- and bcrp- deficient knockout mice. J. Vet. Pharmacol. Ther..

[69] Gao B., Hagenbuch B., Kullak-Ublick G.A., Benke D., Aguzzi A., Meier P.J. (2000). Organic anion-transporting polypeptides mediate transport of opioid peptides across blood-brain barrier. J. Pharmacol. Exp. Ther..

[70] Billington S., Salphati L., Hop C.E.C.A., Chu X., Evers R., Burdette D., Rowbottom C., Lai Y., Xiao G., Humphreys W.G. (2019). Interindividual and Regional Variability in Drug Transporter Abundance at the Human Blood-Brain Barrier Measured by Quantitative Targeted Proteomics. Clin. Pharmacol. Ther..

[71] Annaert P., Ye Z.W., Stieger B., Augustijns P. (2010). Interaction of HIV protease inhibitors with OATP1B1, 1B3, and 2B1. Xenobiotica.

[72] Tupova L., Hirschmugl B., Sucha S., Pilarova V., Székely V., Bakos É., Novakova L., Özvegy-Laczka C., Wadsack C., Ceckova M. (2020). Interplay of drug transporters P-glycoprotein (MDR1), MRP1, OATP1A2 and OATP1B3 in passage of maraviroc across human placenta. Biomed. Pharmacother..

[73] Kis O., Zastre J.A., Ramaswamy M., Bendayan R. (2010). pH dependence of organic anion-transporting polypeptide 2B1 in Caco-2 cells: Potential role in antiretroviral drug oral bioavailability and drug-drug interactions. J. Pharmacol. Exp. Ther..

[74] Hartkoorn R.C., Kwan W.S., Shallcross V., Chaikan A., Liptrott N., Egan D., Sora E.S., James C.E., Gibbons S., Bray P.G. (2010). HIV protease inhibitors are substrates for OATP1A2, OATP1B1 and OATP1B3 and lopinavir plasma concentrations are influenced by SLCO1B1 polymorphisms. Pharmacogenet. Genom..

[75] Karlgren M., Vildhede A., Norinder U., Wisniewski J.R., Kimoto E., Lai Y., Haglund U., Artursson P. (2012). Classification of inhibitors of hepatic organic anion transporting polypeptides (OATPs): Influence of protein expression on drug-drug interactions. J. Med. Chem..

[76] Hubeny A., Keiser M., Oswald S., Jedlitschky G., Kroemer H.K., Siegmund W., Grube M. (2016). Expression of Organic Anion Transporting Polypeptide 1A2 in Red Blood Cells and Its Potential Impact on Antimalarial Therapy. Drug Metab. Dispos..

[77] Xu C., Zhu L., Chan T., Lu X., Shen W., Madigan M.C., Gillies M.C., Zhou F. (2016). Chloroquine and Hydroxychloroquine Are Novel Inhibitors of Human Organic Anion Transporting Polypeptide 1A2. J. Pharm. Sci..

[78] Cao Y.-C., Deng Q.-X., Dai S.-X. (2020). Remdesivir for severe acute respiratory syndrome coronavirus 2 causing COVID-19: An evaluation of the evidence. Travel Med. Infect. Dis..

[79] Jorgensen S.C.J., Kebriaei R., Dresser L.D. (2020). Remdesivir: Review of Pharmacology, Pre-clinical Data, and Emerging Clinical Experience for COVID-19. Pharmacotherapy.

